# VH032 suppresses glioma proliferation by inhibiting the VHL/HIF-1α/VEGF pathway

**DOI:** 10.1016/j.bbrep.2025.102254

**Published:** 2025-09-18

**Authors:** Yi Zhang, Xin-yu Zhu, Jia-rong Gu, Peng Wang, Yu-jun Wen, Shao-zhang Hou, Jing-yu Yang, Jin-hai Gu

**Affiliations:** a Key Laboratory of Craniocerebral Diseases, Ningxia Medical University, Yinchuan, 750004, China; b School of Public Health, Ningxia Medical University, Yinchuan, 750004, China; c Department of Dermatology, People's Hospital of Ningxia Hui Autonomous Region, Yinchuan, 750002, China

**Keywords:** VH032, Glioma, Apoptosis, Proliferation, VHL, HIF-1α, VEGF pathway

## Abstract

**Purpose:**

VH032 is a VHL ligand employed for the recruitment of the von Hippel-Lindau (VHL) protein. Recent studies reveal VHL exhibits significant antitumour effects on various tumor cells. Nevertheless, VH032's impacts on glioma cells remain largely unexplored.

**Methods:**

The study explored VH032's impact on glioma cell lines U87MG and U251 through a series of experimental assessments. The experiments included cell viability assays, wound healing assays and transwell assays. The apoptotic activity of VH032 was determined via flow cytometry. Finally, a xenograft tumor model composed of nude mice was used to confirm the results.

**Results:**

In vitro, the CCK-8 assay indicated that VH032 potently inhibited the proliferation of glioma cells. Wound healing and transwell assays further revealed that the drug significantly impaired glioma cell migration and invasion. Flow cytometry analysis demonstrated an increased rate of apoptosis in the drug-treated cell population. Moreover, the inhibitory effect on the VHL/HIF-1α/VEGF signaling pathway was confirmed, supporting its role in reducing tumour growth. The xenograft mouse study confirmed that VH032 markedly inhibited tumour growth.

**Conclusion:**

These findings suggest that VH032 is a novel and promising chemotherapeutic agent for gliomas, acting through the interference of the VHL/HIF-1α/VEGF signaling pathway to suppress migration and invasion of glioma cells.

## Highlights

• VH032 represents a promising therapeutic candidate for the treatment of glioma, particularly refractory GBM.
• It exerts potent antitumor effects by inhibiting tumor cell proliferation, migration, and invasion through suppression of the VHL/HIF-1α/VEGF signaling pathway, while also inducing apoptosis and G2/M phase cell cycle arrest.
• In vivo studies have demonstrated its efficacy in significantly suppressing tumor growth, highlighting its potential for clinical translation.

## 1 Introduction

Gliomas are tumors that arise from glial cells of the brain, including astrocytes, oligodendrocytes, and ependymal cells [1]. Among the various types of gliomas, glioblastoma (GBM) is recognized as the most prevalent malignant tumor within the adult central nervous system [2], with an annual incidence rate of approximately 6.6 per 100,000 individuals [3]. GBMs are characterized by their high degree of vascularization [4]. Despite significant advancements in biological research and cancer treatment over recent years, our understanding of GBM—a tumor known for its aggressive nature—remains limited [5]; its precise pathogenesis is still not fully understood [6]. In clinical practice, surgical resection serves as the primary treatment modality, often followed by adjuvant therapies such as radiotherapy, chemotherapy, and immunotherapy [7]. However, traditional therapeutic approaches have demonstrated minimal efficacy in enhancing patient prognosis or extending survival times [8]; these approaches are associated with poor overall treatment outcomes and a high recurrence rate [9]. Statistical data indicate that the median survival time for patients diagnosed with malignant glioma ranges from only 12–15 months [10]. Consequently, investigating the mechanisms underlying the occurrence and progression of GBM has become a critical focus within glioma research aimed at identifying novel strategies and targets for effective GBM treatment.

Von Hippel-Lindau (VHL) is an E3 ligase that functions primarily as a tumor suppressor gene and plays a crucial role in cancer prevention [11]. Numerous studies have demonstrated the efficacy of VHL in cancer treatment, with recent findings highlighting its anti-tumor activity across various malignancies, including renal cell carcinoma and ovarian cancer [12,13]. The loss of VHL has also been linked to conditions such as hemangioblastoma and pheochromocytoma [14,15]. However, research on the impact of the VHL gene on human gliomas remains limited. VH032, a novel ligand for VHL [16], has a unique mechanism of action that opens new avenues for tumor therapy. VH032 facilitates precise regulation of the interaction between VHL and HIF-1α by recruiting the VHL protein. The cornerstone of this regulatory effect lies in the ability of VH032 to interact with target protein ligands through linkers to form proteolysis-targeting chimeras (PROTACs) [17], which can serve as multi-target inhibitors.

Therefore, the objective of this study was to systematically investigate the in vitro and in vivo anti-tumor effects of VH032 on glioma cell lines, specifically U87MG and U251, and to elucidate the potential underlying mechanisms.

## 2 Methods

### 2.1 Reagents

VH032 (99.64 % purity) was purchased from MedChemExpress (USA) for this study. It was carefully handled and suspended in dimethyl sulfoxide (DMSO), then diluted with complete cell culture medium so that the final DMSO concentration did not exceed 0.1 %, thereby minimizing side effects.

Dulbecco's modified Eagle's medium (DMEM), phosphate-buffered saline (PBS), and fetal bovine serum (FBS) were purchased from Thermo Fisher (Waltham, MA, USA). A Cell Counting Kit-8 (CCK-8) was purchased from MedChemExpress. Penicillin/streptomycin (100 × ) was purchased from Beijing Solarbio Science & Technology Co., Ltd. (Beijing, China). The BCA Protein Assay Kit, Annexin V-FITC Apoptosis Detection Kit, and Cell Cycle and Apoptosis Analysis Kit were purchased from Jiangsu KeyGEN BioTECH Co., Ltd. (Jiangsu, China). Tris-buffered saline with Tween-20 (TBS-T) was purchased from Wuhan Servicebio Technology Co., Ltd. (Wuhan, China).

Primary antibodies against VHL, VEGF, and HIF-1 alpha were purchased from Proteintech Group, Inc. (Wuhan, China). Primary antibodies against Tubulin beta, as well as secondary antibodies (goat anti-rabbit IgG and goat anti-mouse IgG), were purchased from Affinity Biosciences Ltd (Jiangsu, China). The experimental instruments used included a cell incubator (Thermo Fisher Scientific), an inverted microscope (Olympus Company, Japan), and a freezing centrifuge (ST16R, Thermo).

### 2.2 Cell lines and cell culture

Human U87MG and U251GBM cell lines were cultured in the Key Laboratory of Cranial Diseases, Ningxia Medical University. Both cell lines were cultured in DMEM supplemented with 10 % FBS and 1 % antibiotics: streptomycin and penicillin under a 5 % CO_2_ atmosphere for proper growth and condition.

### 2.3 Cell viability analysis

Cells were plated at 8 × 10^3^ cells per well into a 96-well plate and allowed to adhere at 37 °C overnight. The medium was removed after the cell adherence and was substituted with the indicated concentrations of VH032. At intervals of 12, 24, 36, and 48 h of treatment, 10 μL of CCK-8 solution was added to the wells in such a way that it would not cause bubble formation. After 1 h of incubation, OD was read at 460 nm.

### 2.4 Wound healing assays

Following a seed of U87MG and U251 cell types onto 6-well plates with an 80–90 % confluence of growth. Scraping the surface of the plate vertically with the tip of a 200 μL sterile pipette gun produces a cell wound of similar width. After taking out of the previous medium, fresh serum-free medium with the indicated concentration of VH032 was added. Scratched area images in each group were captured with an inverted microscope at 0 and 24 h. Area quantification of the wounds healing was determined with the aid of ImageJ software.

### 2.5 Cell invasion assays

A mixture of Matrigel matrix and pre-cooled serum-free medium (1:8) was uniformly applied to the surface of the upper chamber of the transwell and incubated overnight. Subsequently, 1 × 10^4^ U87MG and U251 cells, each in 200 μL of serum-free medium, were seeded into the upper chamber. Then, 700 μL of medium containing 20 % FBS and the specified concentration of VH032 was added to the lower chamber. After a 24-h incubation, a cotton swab was used to gently remove any residual matrix and non-invading cells from the upper chamber. The chamber was then treated with 4 % paraformaldehyde for 15 min to fix the cells, air-dried, and stained with 1 % crystal violet for 30 min. Images of the invading cells were obtained with an inverted microscope, and the number of invading cells was quantified.

### 2.6 Cell cycle analysis

After 24 h of treatment with VH032, U87MG and U251 cells were harvested and fixed in 70 % pre-cooled ethanol at −20 °C for at least 24 h. The cells were then stained with propidium iodide (PI) and RNase for 20 min before being analysed by flow cytometry.

### 2.7 Annexin V-FITC/PI double staining assay

An Annexin V-FITC/PI Apoptosis Detection Kit was used to find the effect of VH032 against apoptosis in the cells of the human glioma. Cells U87MG and U251 were inoculated in 6-well plates at a density of 4 × 10^5^. The cells were incubated for 24 h with VH032. The cells were stained with 100 μL 1 × binding buffer, 5 μL PI, and 5 μL Annexin V-FITC for 30 min at room temperature. 400 μL 1 × binding buffer was added and flow cytometry was used to detect the degree of apoptosis in the glioma cells. The apoptotic cells were measured as a percentage using the software FlowJo.

### 2.8 Western blotting analysis

Proteins of U87MG and U251 cells were treated with VH032 for 24 h and then extracted and quantified by the BCA Protein Assay Kit. Equal quantities of protein (40 μg per lane) were resolved on 8–10 % SDS-PAGE gels and transferred to PVDF membranes in a Bio-Rad wet transfer system (Bio-Rad, USA). Afterward, the membranes were blocked with 5 % skim milk powder for 1 h and then further incubated overnight at 4 °C with the appropriate primary antibodies. Thereafter, membranes were incubated with their corresponding secondary antibodies at room temperature for 1.5 h. After washing, the film was exposed, imaged with a gel imaging system, and the grayscale values were measured.

### 2.9 Xenograft tumor model

BALB/c female nude mice, aged 5–6 weeks and weighing 18–20 g, were selected for the experiment and purchased from GemPharmatech Co., Ltd. in Jiangsu, China. The animal housing and tumor-bearing model construction were conducted in an SPF barrier environment at the Experimental Animal Center of Ningxia Medical University. In a sterile condition, each nude mouse was subcutaneously inoculated with 100 μL of U87MG cell suspension containing 1 × 10^7^ cells into the right axilla. Once the tumor size reached approximately 4 mm, the mice were randomly divided into two groups (n = 5 per group). Both experimental and control groups received intraperitoneal injections daily for 21 consecutive days. The experimental group was administered VH032 at a dose of 7.5 mg/kg, while the control group received an equivalent volume of normal saline.

Every three days, the mice were weighed, along with the longest (L) and shortest (S) tumor measurements. According to the tumor volume equation, volume = 0.5 × L × S^2^, tumor volume was determined. The day after measurement, the nude mice were euthanized via cervical dislocation, and tumor tissue was obtained, photographed, and weighed.

The experimental method was approved by the Animal Care and Use Committee of Ningxia Medical University (Approval No. IACUC-NYLAC-2024-097). All efforts were made to minimize pain and reduce the number of animals used in the research.

### 2.10 Statistical analysis

All the experiments' results were drawn from three or more tests done using GraphPad Prism 9.5.1 (GraphPad Software, Inc., La Jolla, CA, USA). Continuous variable data were represented as means ± standard deviations (SDs). The *t*-test was used to compare and analyze the data between the two groups, and one-way ANOVA was used to analyze the differences between multiple groups. Results with P values of <0.05 were considered significant.

## 3 Results

### 3.1 VH032 suppressed glioma cell proliferation

**Fig. 1:**
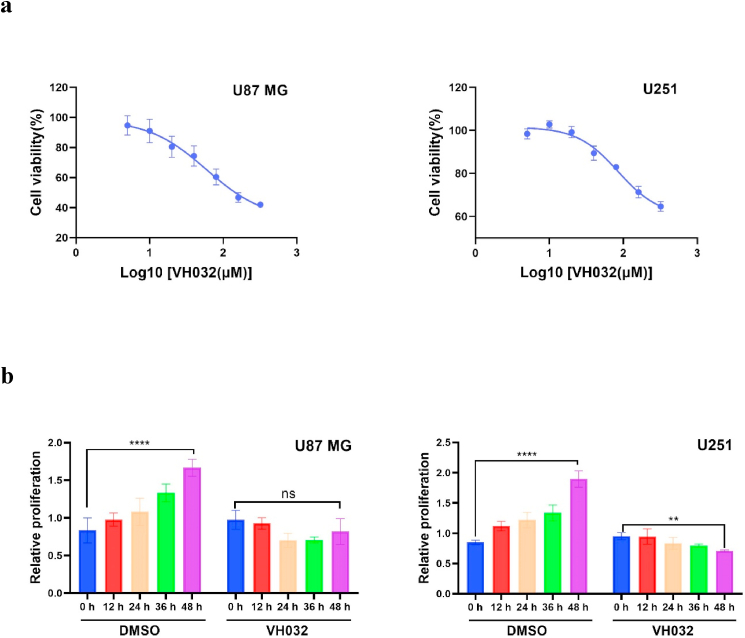
VH032 inhibits the proliferation of glioma cells. a. The viability of U87MG and U251 glioma cells at various concentrations of VH032 was assessed via the CCK-8 assay. **b**. The relative proliferation rates of the migratory capacity of U87MG (treated with 59.2 μM VH032) and U251 (treated with 85.07 μM VH032) cells at different time points were detected by CCK-8.

The effect of VH032 on glioma cell proliferation was examined by treating the cells with varying concentrations of the compound. The results of the CCK-8 assay indicated that as the concentration of VH032 increased within a specific range, the viability of glioma cells decreased in a dose-dependent manner (Fig. 1a). The 24-h half-maximal inhibitory concentration (IC50) values for VH032 against U87MG and U251 cells were determined to be 59.2 μM and 85.07 μM, respectively.

Furthermore, we verified the drug's effect by conducting a CCK-8 cytotoxicity test using VH032 with the measured IC50 concentration, which demonstrated that both glioblastoma U87MG and U251 cells exhibited significant inhibition of proliferation following drug treatment. Notably, this inhibitory effect was observed relatively quickly, beginning approximately 12 h posttreatment, and exhibited a time-dependent nature (Fig. 1b).

### 3.2 VH032 inhibited glioma cell migration and invasion

**Fig. 2:**
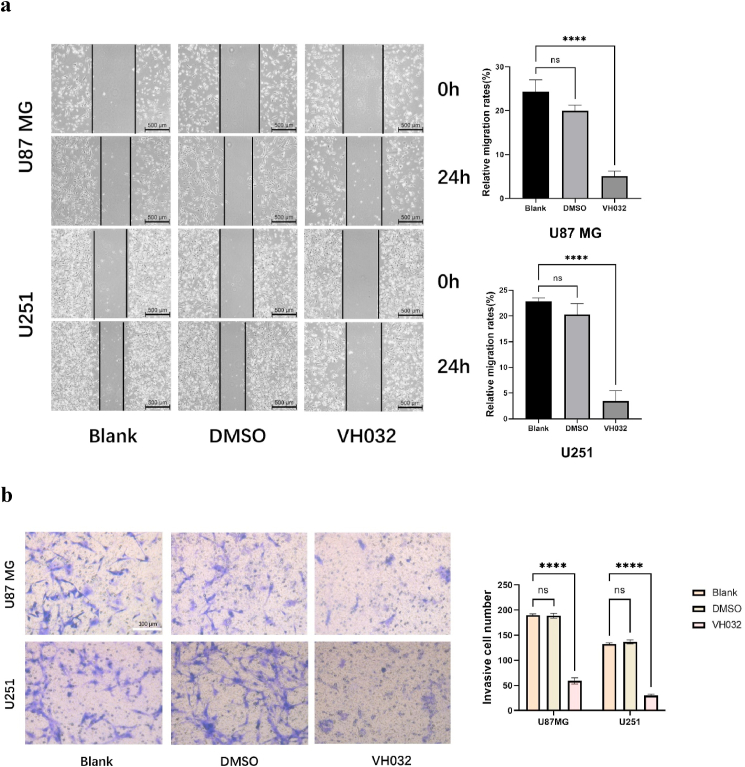
VH032 inhibits the migration and invasion of glioma cells. a. VH032 inhibits the migration of glioma cells in wound healing assay. The migratory capacity of U87MG (treated with 59.2 μM VH032) and U251 (treated with 85.07 μM VH032) cells was evaluated at 0 h and 24 h. b. Transwell assesses were performed using U87MG (59.2 μM VH032) and U251 (85.07 μM VH032) to evaluate the effect of VH032 on glioma cell invasion.

This study investigates VH032's impact on glioma cell migration and invasion through wound healing and transwell assays. The results indicated that VH032 could effectively inhibit wound healing (Fig. 2a) and invasion (Fig. 2b) in both U87MG and U251 cell lines at IC50 concentrations. Specifically, the wound healing rate for the U87MG cell group was 24.36 %. Following a 24-h treatment with VH032, this percentage decreased to 5.12 %. In contrast, the control group of U251 cells presented a wound healing rate of approximately 22.8 %, which decreased to 3.42 % after a similar duration of VH032 treatment. Results from the transwell assay confirmed that after 24 h, the migration of cells from the upper to the lower compartment was much lower in the treatment group than in the control group, highlighting a statistically significant difference between the two.

### 3.3 VH032 caused apoptosis in glioma cells

The process of apoptosis, which refers to programmed cell death, plays a crucial role; evasion of this mechanism allows tumor cells to proliferate indefinitely [18]. Flow cytometry analyses (using 59.2 μM VH032 for U87MG and 85.07 μM VH032 for U251) revealed a significant increase in the number of apoptotic cells during both the early and late stages following VH032 treatment (Fig. 3a).

**Fig. 3a:**
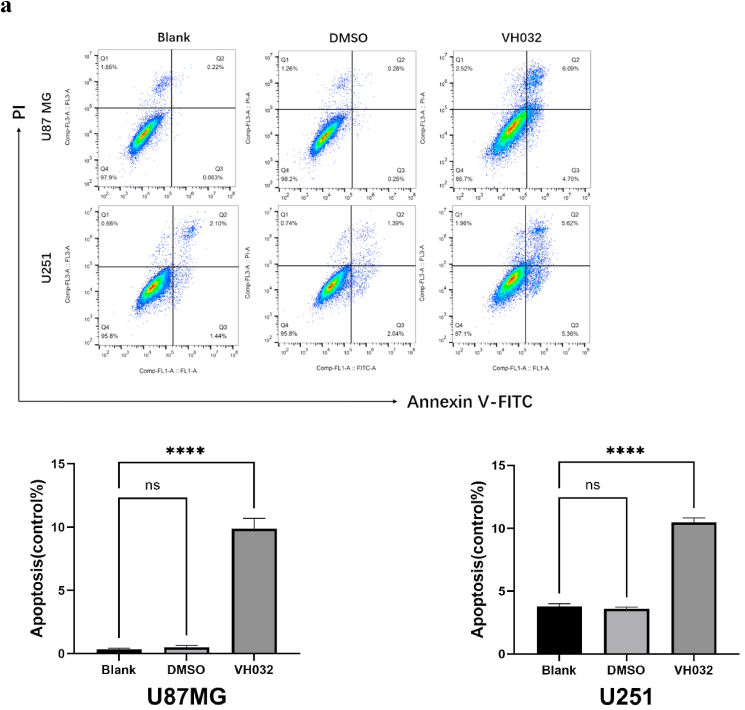
VH032 induced the apoptosis of glioma cells. Flow cytometry analysis of apoptotic U87MG (59.2 μM VH032) and U251 (85.07 μM VH032) cells via Annexin V-FITC/PI staining after 24 h of treatment. The effect of VH032 on the apoptosis of glioma cells was significantly increased.

### 3.4 VH032 induced G2/M phase cell cycle arrest in glioma cells

Flow cytometry is capable of quantifying the DNA content within cells and analysing its distribution across various phases of the mitotic cycle. The data and statistical data indicate that following 24 h of treatment with VH032 (59.2 μM for U87MG and 85.07 μM for U251), there was a notable alteration in the proportion of U87 MG and U251 cells at each phase, with a significant increase in those arrested at the G2/M stage. These findings suggest that VH032 effectively induces G2/M phase arrest in U87 MG and U251 cells (Fig. 3b).

**Fig. 3b:**
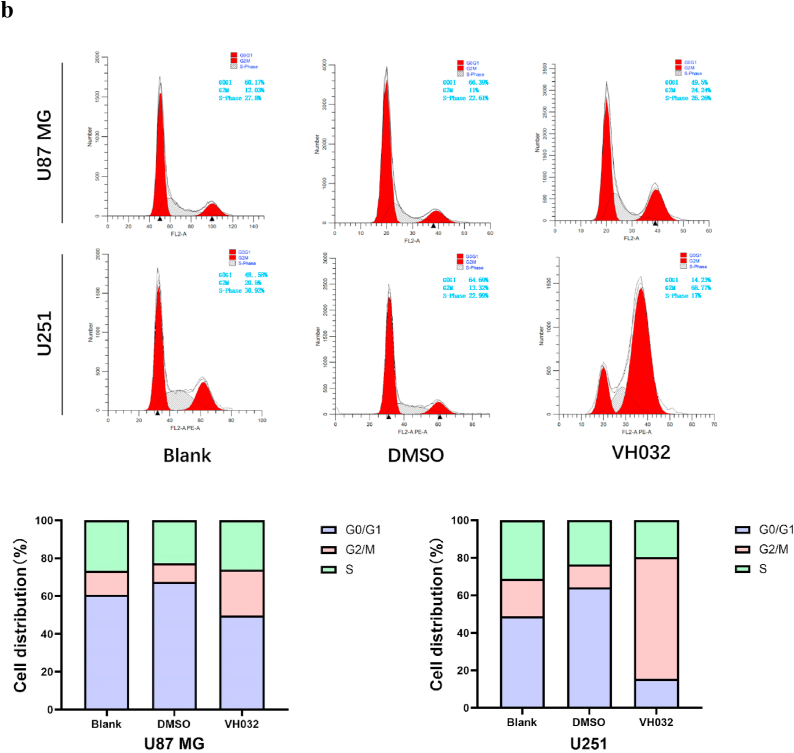
VH032 induced G2/M cell cycle arrest in glioma cells. Flow cytometry analysis of cell cycle distribution in U87MG (59.2 μM VH032) and U251 (85.07 μM VH032) cells after 24 h of treatment, and the proportion of cells in the G2/M phase increased significantly.

#### 3.4.1 VH032 inhibited the VHL/HIF-1α/VEGF signaling pathway in glioma cells

**Fig. 4:**
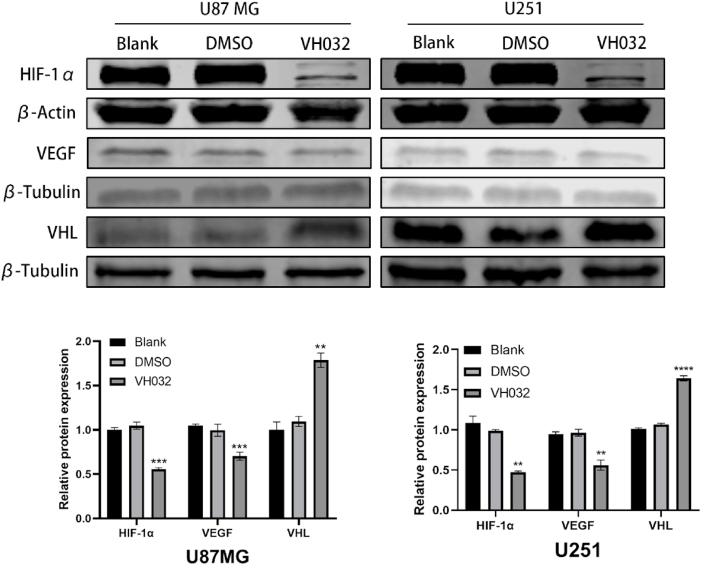
VH032 affects cell viability, migration, invasion and apoptosis by inhibiting the VHL/HIF-1α/VEGF signalling pathway. Western blotting analysis of VHL, HIF-1α, and VEGF protein levels in U87MG (59.2 μM VH032) and U251 (85.07 μM VH032) cells after 24 h of treatment.

The above results demonstrated that VH032 has a significant regulatory effect on tumor migration, invasion and apoptosis. To gain a deeper understanding of the molecular mechanisms at play, we conducted a western blotting analysis. The findings indicated that VH032 (59.2 μM for U87MG and 85.07 μM for U251) had a significant effect in lowering the total protein levels of VHL, HIF-1α, and VEGF (Fig. 4).

### 3.5 VH032 inhibited glioma cell proliferation in vivo

**Fig. 5:**
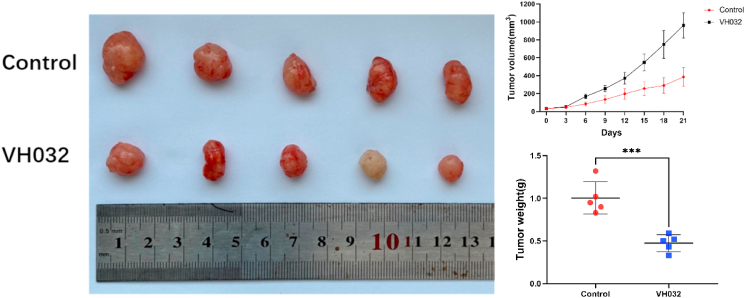
VH032 inhibited tumor growth in xenografted mice. Tumor extraction and imaging were performed in control mice and VH032-treated mice. After 21 days of treatment with VH032, the mean volume and weight of the glioma grafts were decreased.

Throughout the experiment, all the nude mice showed healthy growth, with no signs of infection, weight loss, adverse reactions, or death. Over the course of the experiment, the tumor volume in the nude mice increased progressively. The tumors in the control group were significantly larger than those in the VH032-treated group (7.5 mg/kg, daily intraperitoneal injection for 21 days); in particular, the VH032 group showed a marked decrease in both the average tumor volume and weight (Fig. 5).

## 4 Discussion

The intention of the study was, in particular, to ascertain the effect that VH032 has against the glioblastoma (GBM) development and the proposed cellular mechanisms involved. The data provided reveal that VH032 inhibits the growth of tumors through interference with the VHL, HIF-1α, and VEGF signaling pathways.

Brain tumors are characterized by high incidence and mortality owing to their localized and locally invasive growth, with gliomas being the most common primary brain tumors [19]. So, an individualized multimodal approach that stresses treatment for GBM should be done. Recently, large-scale clinical trials have reported a median survival of about 20.9 months in patients with GBM after surgery, radiotherapy, temozolomide, and treatment against the tumor [20]. The general prognosis for those affected by GBM remains bleak; the current regimens for chemotherapy are poor, with a focus on problems related to drug resistance and adverse effects. Therefore, urgent options to develop effective and feasible therapy for such malignancy are needed.

Recent studies have shown that VHL exhibits antitumour effects in various types of malignancies [21]. However, little is still unknown about the role of VHL in gliomas. VH032 is a ligand for VHL, and to date, there have been few studies that elucidated the anti-glioma effect of VH032. In this study, VH032 induces apoptosis of glioma cells and inhibits the metastatic activity of glioma cells by influencing the protein content of the VHL/HIF-1α/VEGF pathway.

First, the results of the CCK-8 assay indicated that an increase in the VH032 concentration within a specific range led to a decrease in the viability of glioma cells as the dose increased. The efficacy of the drug was further corroborated through CCK-8 cell cytotoxicity experiments, which demonstrated significant inhibition of cell proliferation in U87MG and U251 glioblastoma cells following drug treatment. Additionally, the viability of glioma cells decreased over time. Secondly, the wound healing and transwell assays confirmed the effects of VH032 on cell migration and invasion. The processes of migration and invasion in malignant tumor cells are intricate and multifaceted [22]. Thirdly, Annexin V-FITC/PI dual staining was employed to assess cell apoptosis. These results indicated that VH032 effectively induced apoptosis in these cells. Apoptosis is a form of programmed cell death, and its inhibition can result in the uncontrolled proliferation of tumor cells [23]. In addition, flow cytometry revealed that the proportion of G2/M phase cells in VH032 treatment group was significantly higher than that in the control group.

Additionally, recent studies demonstrated that activation of the VHL/HIF-1α/VEGF pathway relates with tumor cell occurrence, growth, prognosis, and drug resistance^[^ [[24], [25], [26], [27]]^]^. Hypoxia inducible factor-1 (HIF-1) is one of the most important targets and important regulators of tumor angiogenesis since its activation is dependent on hypoxia and promotes tumor cell growth, angiogenesis, and metabolic adaptation [28,29]. VEGF regulates angiogenesis and vascular permeability; this gene has high expression levels in gliomas, associated with their aggressiveness [[30], [31], [32]]. This means that through inhibition of HIF-1α and VEGF, VH032 may reverse or slow down these tumor-related processes. The last proof of the anti-glioma effect of VH032 in vivo was established through a xenograft tumor model in nude mice where U87 MG cells were transplanted. In the cell experiments, the final concentration of DMSO was strictly controlled at ≤0.1 %. The experiments confirmed that this concentration had no significant effect on the proliferation and migration of U87MG and U251 cells (compared with the blank group), indirectly suggesting that the interference of low concentrations of DMSO might be weaker, providing a certain reference for the interpretation of in vivo results. These studies confirmed the inhibitory effect of VH032 on transplanted tumors.

## 5 Conclusions

The results of the present study revealed that VH032 plays a crucial role in antitumour effects on glioma cells in vitro by inhibiting the VHL/HIF-1α/VEGF signalling pathway and interfering with the proliferation, invasion, migration, apoptosis and cycle distribution of glioma cells. Taken together, these findings provide evidence that VH032 is a potential therapeutic agent for glioma.

## CRediT authorship contribution statement

**Yi Zhang:** Writing – review & editing, Writing – original draft, Methodology, Data curation. **Xin-yu Zhu:** Methodology. **Jia-rong Gu:** Validation. **Peng Wang:** Visualization. **Yu-jun Wen:** Supervision. **Shao-zhang Hou:** Validation. **Jing-yu Yang:** Resources, Funding acquisition, Conceptualization. **Jin-hai Gu:** Writing – review & editing, Funding acquisition, Conceptualization.

## Ethics approval and consent to participate

Approval of the research protocol by an institutional review board: The research protocol was approved by the Institutional Review Board of Ningxia Medical University(Approval no. IACUC-NYLAC-2024-097).

Animal Studies: All animal procedures and experimental methods were approved by the Medical Ethics Committee of Ningxia Medical University.

## Clinical trial number

Not applicable.

## Data availability

The data used to support the findings of this study are included within the article.

## Funding

The author(s) disclosed receipt of the following financial support for research, authorship, and/or publication of this article: This work was supported by the 10.13039/501100004772Natural Science Foundation of Ningxia (grant nos. 2022AAC03357, 2020AAC03152, 2021AAC03121) and the 10.13039/501100001809National Natural Science Foundation of China (grant no. 82260269).

## Declaration of competing interest

The authors declare that they have no competing interests.

## Data Availability

Data will be made available on request.
