# Supplementary figures and images for: VH032 suppresses glioma proliferation by inhibiting the VHL/HIF-1α/VEGF pathway
Source: Biochem Biophys Rep. 2025 Sep 18;44:102254. doi: 10.1016/j.bbrep.2025.102254 (PMC12477846; doi:10.1016/j.bbrep.2025.102254)

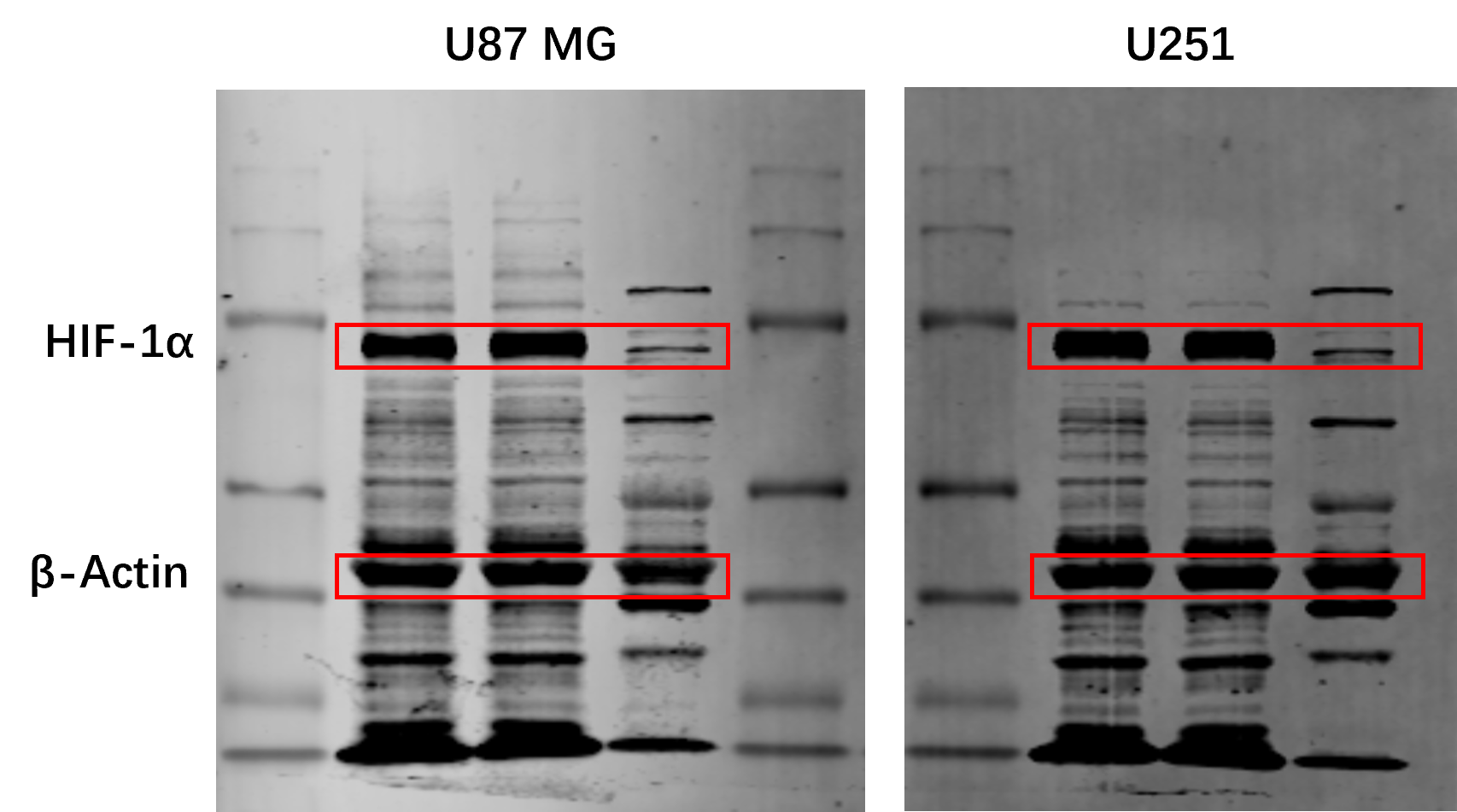

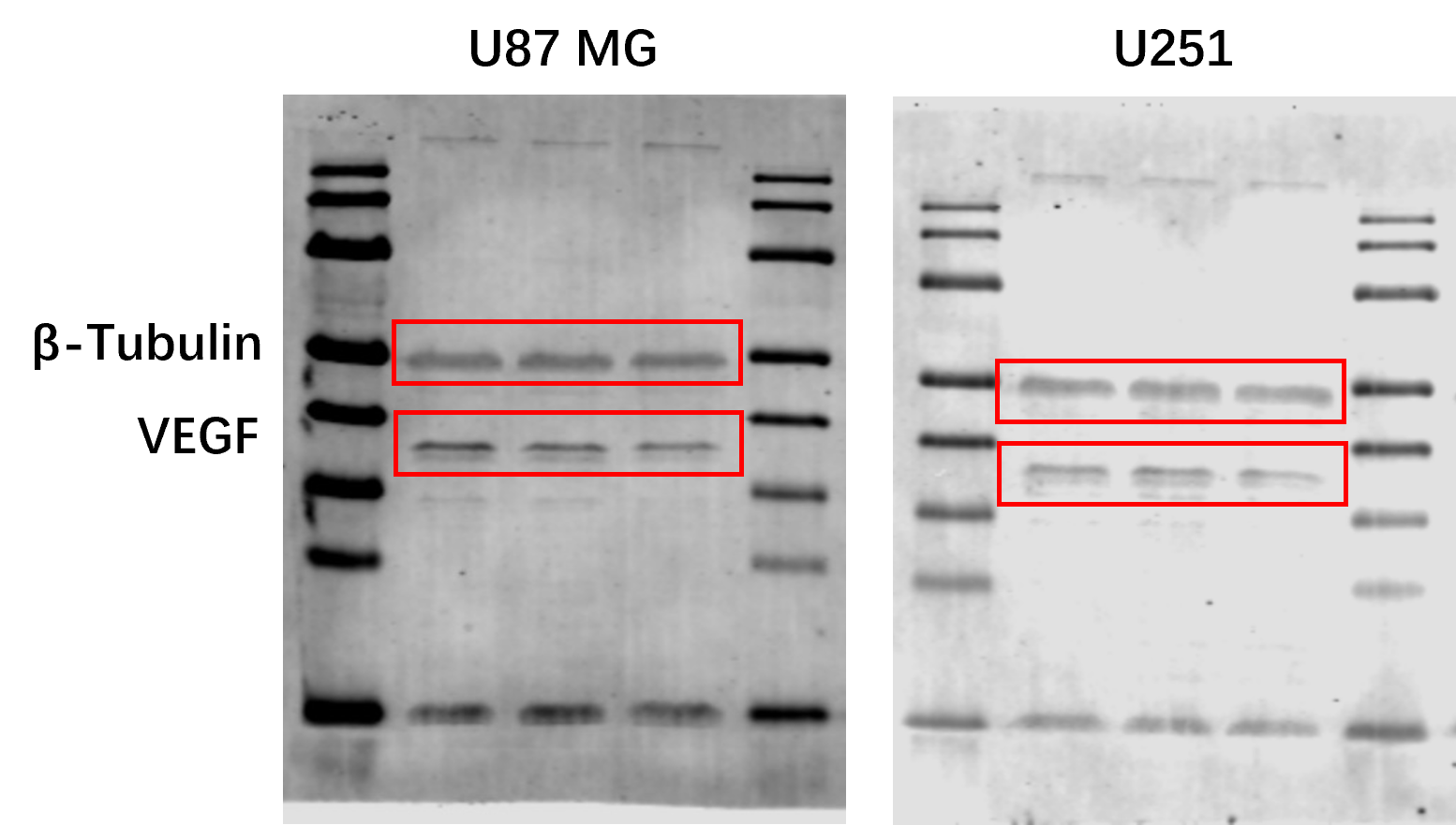

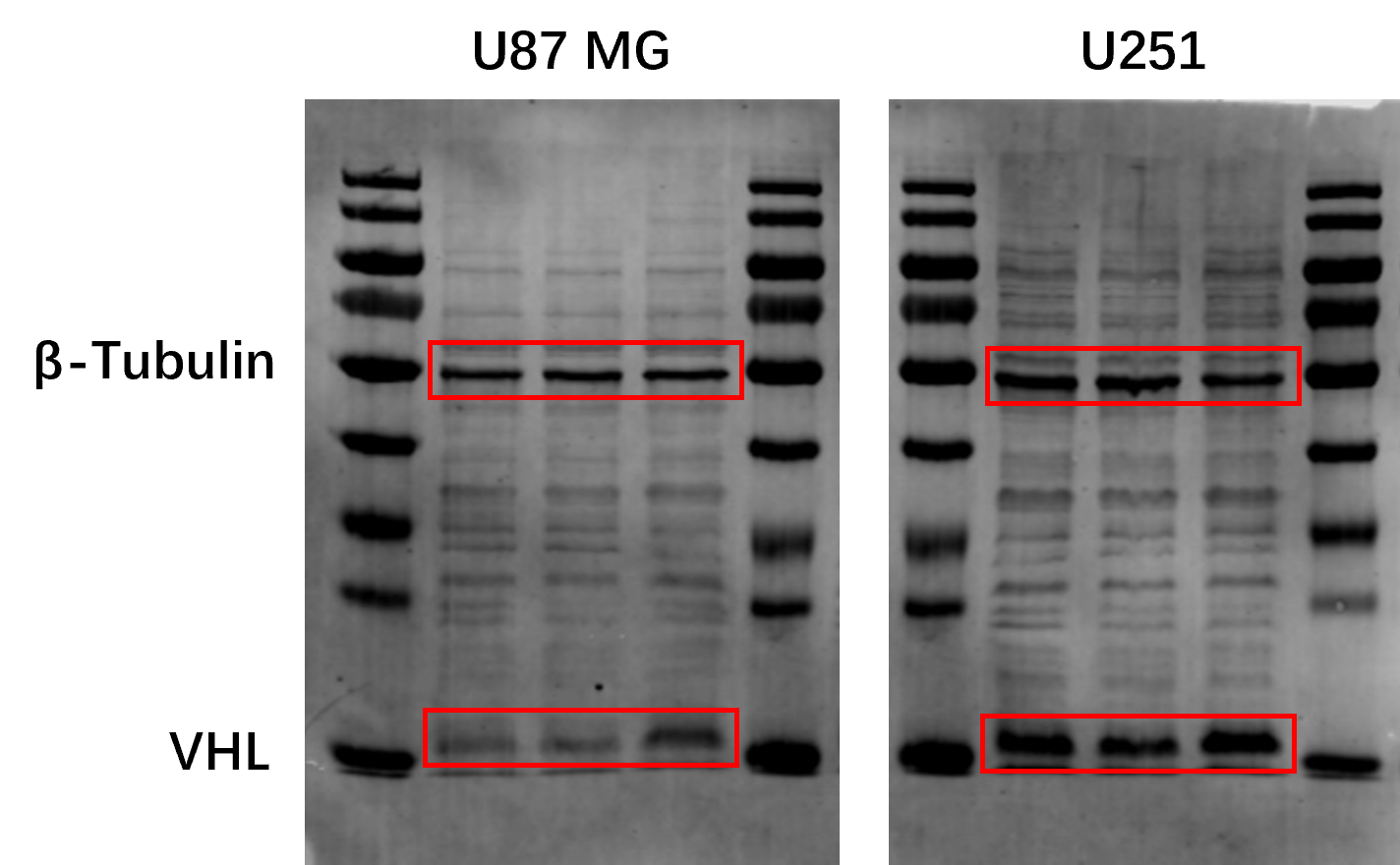

Supplement: Multimedia component 1 [file mmc1.docx]
